# Risk Factors Associated with Postoperative Nausea and Vomiting After Esophagogastroduodenoscopy

**DOI:** 10.3390/healthcare14101340

**Published:** 2026-05-14

**Authors:** Gülencan Yumuşak Ergin, Hazal Ekin Guran Aytuğ, Mustafa Ergin

**Affiliations:** 1 Department of Anesthesiology and Reanimation, Aksaray University Training and Research Hospital, 68200 Aksaray, Türkiye; 2 Department of Anesthesiology and Reanimation, Etlik City Hospital, 06170 Ankara, Türkiye; drekinguran@gmail.com; 3 Department of Gastroenterology, Aksaray University Training and Research Hospital, 68200 Aksaray, Türkiye; mstfergn@hotmail.com

**Keywords:** postoperative nausea and vomiting, esophagogastroduodenoscopy, Apfel score

## Abstract

**Highlights:**

**What are the main findings?**

- The incidence of postoperative nausea and vomiting (PONV) after esophagogastroduodenoscopy (EGD) performed under sedation was 8.6%, and female sex, higher body mass index (BMI), preoperative nausea, thyroid disease, and oral antidiabetic drug use were significantly associated with increased risk.
- Higher Apfel scores were associated with the development of PONV, whereas macroscopic endoscopic findings and sedative agents were not significantly related to its occurrence.

**What are the implications of the main findings?**

- Preoperative evaluation of patient-related risk factors such as sex, BMI, thyroid disease, oral antidiabetic drug use, and Apfel score may help identify individuals at higher risk for PONV before EGD procedures.
- Improved awareness of these risk factors may contribute to better perioperative management and postoperative comfort in patients undergoing endoscopic procedures.

**Abstract:**

**Background/Objectives**: Postoperative nausea and vomiting (PONV) is a common complication that may negatively affect patient comfort and recovery following procedures performed under sedation. Although gastrointestinal endoscopic procedures are widely performed, data on the incidence and risk factors of PONV after esophagogastroduodenoscopy (EGD) remain limited. This study aimed to determine the incidence of PONV following EGD under sedation and to identify factors associated with its development. **Methods**: This single-center retrospective study included adult patients who underwent elective EGD under sedation between June and November 2023. Demographic and clinical data, Apfel risk scores, sedative agents, procedure duration, and macroscopic endoscopic findings were obtained from electronic medical records. PONV was assessed based on documentation during the post-anesthesia care unit stay. Patients were categorized into PONV-positive and PONV-negative groups and compared using appropriate statistical tests. **Results**: A total of 152 patients were included, and PONV occurred in 13 patients (8.6%). Female sex (*p* = 0.020), higher body mass index (BMI) (*p* = 0.009), preoperative nausea or vomiting (*p* = 0.002), thyroid disease (*p* = 0.004), oral antidiabetic drug use (*p* = 0.003), and higher Apfel risk scores (*p* = 0.008) were significantly associated with PONV. Age, American Society of Anesthesiologists (ASA) score, procedure duration, sedative agents, and macroscopic endoscopic findings showed no significant association. **Conclusions**: PONV following EGD under sedation was relatively uncommon. Patient-related factors, particularly female sex, higher BMI, preoperative nausea, thyroid disease, oral antidiabetic drug use, and higher Apfel scores, were associated with increased risk.

## 1. Introduction

Nausea, vomiting, and retching after surgery are frequent issues that can negatively impact a patient’s recovery from anesthesia. These symptoms are often perceived as distressing by patients and may compromise both their postoperative comfort and overall recovery experience. Postoperative nausea and vomiting (PONV) can lead to longer hospital stays, higher medical expenses, repeat hospital visits, and rare but serious complications like aspiration pneumonia [1,2,3]. PONV remains a significant concern in anesthetic practice due to its undesirable effects on patient comfort and postoperative recovery. Although it is often self-limiting or can be managed effectively without long-term complications, considerable research has focused on determining its incidence and associated risk factors [4]. Many patients would prefer pain over PONV and would be willing to absorb the additional cost to avoid PONV. As the need for ambulatory surgery increases, the prevention of PONV continues to become more important to the system-based approach of anesthesiology [5,6]. The likelihood of developing PONV is influenced by multiple variables, including the type of surgical procedure, the anesthetic technique, and individual patient characteristics.

While PONV affects nearly one-third of surgical patients, its incidence can reach up to 80% among individuals with multiple risk factors [7]. Various interventions have been explored in the literature to alleviate perioperative nausea and vomiting, aiming to decrease its frequency, severity, and persistence [8]. Widely used in clinical settings, the Apfel score evaluates PONV risk based on four factors: gender, smoking status, history of PONV or motion sickness, and postoperative opioid use [9]. Risk assessment tools similar to the Apfel score also incorporate additional variables such as age and procedure duration [10]. Procedure-related risk factors are suggested to play a role in PONV. The likelihood of PONV is particularly elevated after procedures such as laparoscopic surgeries, bariatric operations, gynecologic interventions, and cholecystectomy [11].

Gastrointestinal endoscopic procedures may lead to nausea and vomiting as a result of overstretching the stomach or intestinal segments during the intervention [12]. Previous studies have reported varying rates of nausea and vomiting following gastrointestinal endoscopic procedures [13,14]. The incidence of nausea and vomiting after gastrointestinal endoscopic procedures has been reported to be 1.42% [14], with PONV identified as the third most common complication, following hemodynamic instability and dysrhythmia. Endoscopic procedures are widely performed globally, and PONV represents a common and potentially serious complication; however, its true incidence and contributing risk factors following these procedures have yet to be clearly defined.

Therefore, the primary aim of this study is to determine the incidence of PONV following esophagogastroduodenoscopy (EGD) performed under sedation. The secondary aim was to identify patient-, procedure-, and endoscopy-related factors associated with the development of PONV, including the potential impact of macroscopic endoscopic findings.

## 2. Materials and Methods

### 2.1. Study Design and Patient Selection

This single-center, retrospective study was conducted at the gastroenterology unit of Aksaray University Training and Research Hospital. Patients classified as American Society of Anesthesiologists (ASA) physical statuses I–III who underwent elective EGD under sedation between June and November 2023 were screened for eligibility.

**Figure 1:**
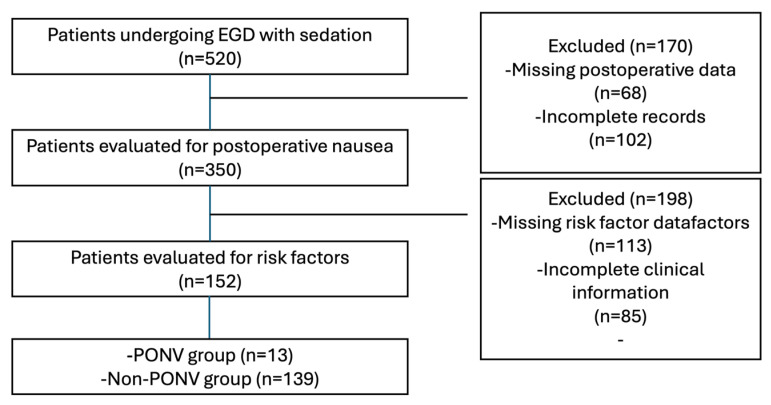
Flow diagram of patient selection and study inclusion.

During the study period, a total of 520 patients underwent EGD under sedation and were initially reviewed. PONV assessment was available in 350 patient records as part of routine postoperative monitoring in the post-anesthesia care unit (PACU). In routine clinical practice, documentation of PONV after endoscopic procedures is not standardized and may vary among healthcare personnel. Complete perioperative and postoperative data were available for 152 patients, who constituted the final study cohort. Patients with missing or incomplete documentation regarding postoperative nausea assessment or key perioperative variables were excluded from the analysis (Figure 1).

All endoscopic procedures were performed by a single experienced gastroenterologist, ensuring procedural consistency and minimizing operator-related variability. During the study period, sedation was administered by a single anesthesiologist (the corresponding author), as no other anesthesiology staff were involved in endoscopic procedures at that time. All eligible patients were included based on predefined inclusion and exclusion criteria, without selection based on outcomes. All patients underwent routine pre-procedural anesthesia evaluation in the outpatient setting. On the day of the procedure, patients were monitored using standard ASA monitoring, including non-invasive blood pressure, heart rate, oxygen saturation, and continuous electrocardiography. Supplemental oxygen was administered via nasal cannula at 6 L/min. Sedation was induced using a predominantly propofol-based regimen. Propofol (1–2 mg/kg) was administered for induction, followed by additional boluses (0.5 mg/kg) as needed to maintain adequate sedation. Additional sedative or analgesic agents were administered at the discretion of the anesthesiologist based on patient-specific factors such as anxiety and discomfort.

#### 2.1.1. Ethic and Trial Registration

This study was conducted in accordance with the Declaration of Helsinki and approved by the Human Ethics Review Committee of Aksaray University Hospital (approval number:2023/23-37 date: 7 December 2023). It was also registered with ClinicalTrials.gov (ID: NCT06689046 date: 11 October 2024). As this was a retrospective study, the requirement for additional informed consent for participation was waived. However, written informed consent had been routinely obtained from all patients prior to the endoscopic procedure as part of standard clinical practice.

#### 2.1.2. Data Collection

Demographic and clinical data, including age, sex, weight, height, body mass index (BMI), ASA physical status, comorbidities, medications, smoking status, alcohol consumption, history of PONV, history of motion sickness, and Apfel risk score were retrieved from electronic medical records. Procedural data included sedative agents administered during EGD and macroscopic endoscopic findings recorded during the procedure.

#### 2.1.3. Assessment of PONV

PONV was evaluated based on medical records documented during the immediate postoperative period while patients were monitored in the PACU. Assessments recorded until patients achieved a modified Aldrete score ≥9 and met institutional discharge criteria were reviewed. The administration of antiemetic medications during this period was also recorded.

PONV was defined as patient-reported nausea and/or documented vomiting during the PACU stay. Due to the retrospective design, no validated severity scoring system was consistently used, and symptoms were recorded in a binary manner based on routine clinical documentation. Postoperative pain scores, documented using the visual analog scale (VAS) during the same monitoring period, were also reviewed.

#### 2.1.4. Statistical Analysis

Statistical analyses were performed using SPSS version 29.0 (IBM Corp., Armonk, NY, USA). Categorical variables are presented as counts and percentages, while continuous variables are summarized as the mean ± standard deviation or median (range), as appropriate. Group comparisons were conducted using the Chi-square or Fisher exact test for categorical variables and Student’s *t*-test or the Mann–Whitney U test for continuous variables. A *p*-value < 0.05 was considered statistically significant.

This study was reported in accordance with the STROBE (Strengthening the Reporting of Observational Studies in Epidemiology) guidelines.

## 3. Results

A total of 152 patients were included in this study (*n* = 152). The demographic data of the patients are presented in Table 1.

In this study, the macroscopic diagnoses observed among the participants were as follows: pangastritis was the most prevalent, found in 79 cases (52.0%). Gastroesophageal reflux was the second most common diagnosis, identified in 32 patients (21.1%). A total of 14 patients (9.2%) had peptic ulcer. Hiatal hernia was present in 6 cases (3.9%), and alkaline reflux gastritis was diagnosed in 7 patients (4.6%). Esophageal varices were observed in 6 cases (3.9%). Less common diagnoses included antral gastritis in 2 patients (1.3%), celiac disease in 4 patients (2.6%), malignancy in 1 patient (0.7%), and candida esophagitis in 1 patient (0.7%) (Table 2). Regarding anesthetic medications administered, propofol was the most frequently used agent, given to 151 patients (99.3%). Midazolam was used in 34 cases (22.4%), while fentanyl was administered in 13 cases (8.6%). A smaller number of patients received remifentanil (2 cases, 1.3%) and ketamine (1 case, 0.7%) (Table 2).

Among preoperative factors, nausea or vomiting was reported in 27 patients (17.8%). Of the total study cohort, 13 patients (8.6%) experienced PONV, while 139 patients (91.4%) did not. Only 2 patients (1.3%) required antiemetic treatment (Table 3).

Demographic, clinical, and perioperative variables were compared between the groups (Table 4). The mean age was 51.6 ± 12.2 years in the PONV group and 44.5 ± 14.4 years in the non-PONV group, with no significant difference (*p* = 0.088). All patients who experienced PONV were female, whereas 69.8% of those who did not experience PONV were female (*p* = 0.020). The median weight for those who experienced PONV was 77 kg (range 54–105 kg), compared to a median weight of 73 kg (range 40–115 kg) in those who did not experience PONV, with no statistically significant difference (*p* = 0.239). Similarly, the median height was 160 cm (range 145–165 cm) in the PONV group and 163 cm (range 145–194 cm) in the non-PONV group (*p* = 0.027). Median BMI was higher in the PONV group than in the non-PONV group (31.6 [22.5–42.0] vs. 26.6 [16–51], *p* = 0.009). When BMI was analyzed as a categorical variable (BMI < 30 vs. ≥30), patients with BMI ≥ 30 had a significantly higher incidence of PONV compared to those with BMI < 30 (20.0% vs. 3.7%, *p* = 0.002). This corresponds to an approximately sixfold increased risk of PONV in patients with BMI ≥ 30.

The ASA score was similar between the two groups, with a median score of 2 (range 1–3) in both groups (*p* = 0.287). The association between comorbidities and PONV was also evaluated. Diabetes mellitus showed a borderline association with PONV (*p* = 0.056). No significant associations were observed between PONV and hypertension, coronary artery disease, pulmonary disease, chronic kidney disease, cirrhosis, insulin use, or antihypertensive drug use (*p* > 0.05 for all). Thyroid disease was significantly associated with an increased incidence of PONV (*p* = 0.004). Similarly, the use of oral antidiabetic drugs was also associated with a higher incidence of PONV (46.2% vs. 10.8%, *p* = 0.003). When oral antidiabetic drugs were analyzed according to specific classes, metformin use was significantly associated with a higher incidence of PONV (27.8% vs. 6.0%, *p* = 0.009). Similar trends were observed for DPP-4 inhibitors and SGLT-2 inhibitors, although these did not reach statistical significance.

Postoperative VAS scores were similar between the groups, with a median of 0 in both (*p* = 0.087). Smoking was more frequent in patients without PONV (28.8% vs. 15.4%), with no significant difference (*p* = 0.517). Alcohol consumption was reported in 5.0% of the non-PONV group, whereas none of the patients in the PONV group consumed alcohol (*p* = 1.000). A history of PONV was present in 30.8% of patients who experienced PONV, compared to 10.8% of those who did not (*p* = 0.060). Motion sickness was reported by 30.8% of patients in the PONV group and 20.1% of those in the non-PONV group, with no significant difference (*p* = 0.474). The Apfel score, a well-established risk score for PONV, was significantly higher in patients who experienced PONV. In the PONV group, 84.6% had an Apfel score of 2, compared to 46.8% in the non-PONV group (*p* = 0.008). Other Apfel score distributions were similar between the groups.

Preoperative nausea or vomiting was reported more frequently in the PONV group than in the non-PONV group (53.8% vs. 14.4%, *p* = 0.002). Macroscopic diagnoses were similar between the two groups, with no significant differences in the types of gastric conditions observed. Pangastritis was the most common diagnosis in both groups, occurring in 53.8% of the PONV group and 51.8% of the non-PONV group. Rates of gastroesophageal reflux, hiatal hernia, and alkaline reflux gastritis were similar between the groups (*p* = 0.561). There was no significant difference between the two groups in terms of the medications administered. Propofol was used in all patients in the PONV group and 99.3% of patients in the non-PONV group (*p* = 1.000). Other medications like midazolam, fentanyl, and remifentanil were administered at similar rates across both groups, with no significant differences. There was no statistically significant difference between patients with and without PONV in terms of the sedative agents administered and their recorded dosages (*p* > 0.05). The duration of the endoscopic procedure was similar between patients with and without PONV (median 7 [7–8] vs. 7 [6–8] min, *p* = 0.73). Due to the limited number of PONV events and the presence of complete separation for female sex (all patients with PONV were female), a reliable multivariate logistic regression analysis could not be performed.

## 4. Discussion

Patients undergoing gastroesophageal interventions often present with symptoms such as nausea and vomiting [12]. In this study population, PONV occurred in 8.6% of patients undergoing EGD under sedation. Previous studies have reported a wide range of PONV incidence after painless digestive endoscopy, varying between 3.3% and 33.3%, depending on patient-related and procedural factors [13,14]. This variability underscores the multifactorial nature of PONV and the challenges associated with its prediction in endoscopic practice.

Variations in PONV rates are influenced by numerous aspects, such as demographic traits (e.g., age, sex, and past motion sickness), anesthetic choices, and the nature and duration of the procedure itself [15].

Female sex was found to be significantly associated with an increased risk of PONV. All patients who experienced PONV in our cohort were female, consistent with the well-established role of sex as a major risk factor for PONV. Hormonal influences, differences in visceral sensitivity, and psychosocial factors have been proposed as potential explanations for this association [16]. The absence of PONV among male patients should be viewed cautiously, considering the limited number of male participants and the imbalance in sex distribution.

An additional important observation was the association between higher BMI and the development of PONV. Patients who experienced PONV had significantly higher BMI values compared with those who did not. Furthermore, when BMI was analyzed as a categorical variable, patients with BMI ≥ 30 had a significantly higher incidence of PONV compared to those with BMI < 30, suggesting a possible threshold effect.

Existing evidence regarding the association between BMI and PONV is inconsistent. While some studies suggest that increased BMI may predispose patients to PONV due to factors such as delayed gastric emptying, increased intra-abdominal pressure, and altered pharmacokinetics of anesthetic agents [17], others have reported a protective effect of higher BMI [18], and some studies argue that BMI should not be considered a risk factor for PONV [19,20]. Our findings contribute to this ongoing debate and suggest that BMI may be associated with an increased risk of PONV following EGD under sedation, although further prospective studies are required to clarify this relationship. A statistically significant difference in height was found between patients with and without PONV; however, the clinical relevance of this finding remains unclear and may be related to its association with BMI rather than height itself.

In this study, both thyroid disease and the use of oral antidiabetic drugs were associated with PONV. In our cohort, thyroid disease mainly reflected patients receiving thyroid hormone replacement therapy, as individuals with overt thyroid dysfunction are typically not scheduled for elective procedures until a euthyroid state is achieved.

Thyroid dysfunction has been reported to affect gastrointestinal motility and metabolic regulation, which may increase susceptibility to symptoms such as nausea. Both hypothyroidism and hyperthyroidism may disrupt hormonal homeostasis and contribute to gastrointestinal dysmotility through mechanisms involving bile acid metabolism, autoimmunity, and altered neurohormonal regulation. Impaired gastrointestinal motility may manifest as nausea, vomiting, dyspeptic symptoms, and small intestinal bacterial overgrowth. These mechanisms may partly explain the observed association between thyroid disease and an increased incidence of PONV [21,22].

When oral antidiabetic drugs were analyzed according to specific classes, metformin use was significantly associated with an increased incidence of PONV. Similar trends were observed for DPP-4 inhibitors and SGLT-2 inhibitors; however, these findings did not reach statistical significance, likely due to the small sample size. No cases of PONV were observed among patients using sulfonylureas or TZDs, although the limited number of patients precludes meaningful conclusions.

The association between metformin use and PONV may be related to the well-recognized gastrointestinal adverse effects of metformin, including nausea, vomiting, abdominal discomfort, and diarrhea. Although the exact mechanisms underlying metformin-related gastrointestinal symptoms are not fully understood, alterations in gastrointestinal motility, gut microbiota, and intestinal serotonin signaling have been proposed [23,24]. Interestingly, metformin use was not strongly associated with preoperative nausea in our cohort, suggesting that the relationship between metformin and PONV may not be fully explained by baseline gastrointestinal symptoms alone. Furthermore, due to the retrospective design, information regarding the timing of antidiabetic medication use, including whether the drugs were taken on the day of the procedure, was not consistently available.

The Apfel risk score, one of the most widely used tools for predicting PONV, was significantly higher in patients who experienced PONV in this study. In addition, the individual components of the score were analyzed separately, and only female sex was significantly associated with PONV, whereas the other components were not. Despite this, the overall Apfel score appeared to be associated with an increased risk of PONV.

However, these findings should be interpreted with caution, as the Apfel score was originally developed and validated in surgical populations undergoing general anesthesia. The incidence and risk profile of PONV may differ between general anesthesia and sedation-based procedures, particularly considering the known antiemetic properties of propofol [6]. Therefore, the applicability of the Apfel score in patients undergoing EGD under sedation remains uncertain and requires further prospective validation.

In the present study, preoperative nausea and/or vomiting was reported in 17.8% of patients; however, most of these patients did not develop postoperative symptoms. This observation may partly be related to the widespread use of propofol-based sedation in our cohort, given the well-established intrinsic antiemetic properties of propofol [17,25,26]. In contrast to volatile anesthetics and opioid-based techniques, which are known to increase the risk of PONV, propofol-based sedation is generally associated with a lower incidence of PONV. In addition, no significant association was identified between the use of supplementary sedative agents or their recorded dosages and the occurrence of PONV. However, because non-propofol anesthetic regimens were rarely used in our cohort, direct comparisons between propofol- and non-propofol–based techniques could not be performed. Furthermore, the relatively uniform use of propofol-based sedation may have reduced variability in anesthetic exposure, thereby limiting the ability to detect differences related to anesthetic techniques.

These findings suggest that greater attention to patient-related risk factors, particularly preoperative nausea and established risk scores such as the Apfel score, may help identify patients at increased risk for PONV following EGD under sedation. Increased awareness of PONV risk in susceptible patients may contribute to improved postoperative comfort and recovery.

In addition to patient-related risk factors, this study explored the potential association between macroscopic endoscopic findings and the development of PONV. Although conditions such as gastritis, gastroesophageal reflux disease, peptic ulcer disease, and hiatal hernia may theoretically increase visceral sensitivity or gastric irritation, no significant association was observed between macroscopic endoscopic findings and PONV in our cohort. This finding suggests that patient-related factors may play a more prominent role in PONV development than endoscopic findings alone. Since histopathological diagnosis and *H. pylori* status would not alter the preoperative approach, these variables were not included in the study.

Although often considered a minor complication, PONV may negatively affect patient comfort, delay discharge, and reduce overall patient satisfaction following endoscopic procedures. In routine clinical practice, PONV following gastrointestinal endoscopic procedures may also be under-recognized and under-documented, particularly when symptoms are mild or transient. The retrospective nature of this study likely reflects this real-world limitation and may partly explain the incomplete availability of systematic PONV assessments. These findings highlight the need for increased awareness and more standardized documentation of postoperative symptoms in endoscopic units.

### Limitations

Several limitations of this study should be acknowledged. First, the retrospective design inherently limited data completeness and may have introduced selection bias. A substantial proportion of patients was excluded because of incomplete data (520 → 152), which may limit the generalizability of the findings. However, this likely reflects variability in routine clinical documentation rather than systematic exclusion based on patient outcomes. In routine endoscopic practice, documentation of postoperative symptoms and patient-related PONV risk factors is not always standardized, particularly in procedures performed under sedation. Therefore, the high rate of incomplete data observed in this study may also reflect real-world limitations in the routine assessment and documentation of PONV-related risk factors in endoscopic units. In addition, PONV was recorded as a binary outcome without standardized severity grading, which may have contributed to underreporting and variability in symptom assessment.

Second, postoperative follow-up was limited to the immediate recovery period in the PACU, and delayed PONV occurring after discharge within the first 24 h was not evaluated. Therefore, the reported incidence likely reflects early PONV and may underestimate the true overall incidence. This limitation is clinically relevant, as post-discharge nausea and vomiting (PDNV) is increasingly recognized as an important component of postoperative recovery [11]. Female sex, younger age, prior history of PONV, opioid use in the PACU, and nausea during PACU stay have all been reported as important risk factors for PDNV. These findings further support the importance of extended follow-up in future studies evaluating PONV after endoscopic procedures.

Third, preoperative fasting duration and hydration status were not analyzed, although both factors may influence the development of PONV. Due to the retrospective design and lack of standardized documentation, these variables could not be reliably assessed.

Furthermore, the relatively small number of PONV events and the presence of complete separation, with all PONV cases occurring in female patients, limited the ability to perform a reliable multivariate analysis. The imbalance in sex distribution may also restrict the generalizability of the findings.

Despite these limitations, the present study provides clinically relevant real-world data regarding factors associated with PONV following sedated EGD. In addition to established risk factors such as female sex, higher BMI, preoperative nausea, and higher Apfel scores, thyroid disease and oral antidiabetic drug use—particularly metformin use—were also associated with an increased incidence of PONV in our cohort. These findings may help clinicians identify patients at increased risk and consider targeted preventive strategies before endoscopic procedures are performed under sedation. Future prospective studies with standardized postoperative symptom assessment and extended follow-up are needed to further clarify risk factors and management strategies for PONV following endoscopic procedures.

## 5. Conclusions

This study identified several factors associated with PONV following EGD performed under sedation. Female sex, higher BMI, preoperative nausea, thyroid disease, oral antidiabetic drug use, and higher Apfel scores were significantly associated with an increased risk of PONV, whereas macroscopic endoscopic findings did not appear to influence its occurrence.

These findings suggest that greater attention to patient-related risk factors during the preoperative evaluation may contribute to improved patient comfort following EGD under sedation. Although routine prophylactic antiemetic use was not specifically evaluated, heightened awareness of PONV risk in susceptible patients may help clinicians identify individuals who may benefit from closer perioperative monitoring and preventive strategies in clinical practice.

As this study focused exclusively on upper gastrointestinal endoscopy, future prospective studies with standardized postoperative symptom assessment and extended follow-up, as well as comparisons between upper and lower gastrointestinal endoscopic procedures such as colonoscopy, may further improve risk prediction and management strategies for PONV.

## Figures and Tables

**Table 1:** The demographic and clinical characteristics of the participants.

Patient Characteristics	
Age (years) (mean ± SD)	45.1 ± 14.3
Gender, *n* (%)	
Female	110 (72.4)
Male	42 (27.6)
Weight (kg) [median (min–max)]	74 (40–115)
Height (cm) [median (min–max)]	163 (145–194)
BMI [median (min–max)]	26.8 (16–51.1)
ASA score [median (min–max)]	2 (1–3)
Smoking status, *n* (%)	42 (27.6)
Alcohol status, *n* (%)	7 (4.6)
History of PONV, *n* (%)	19 (12.5)
Motion sickness, *n* (%)	32 (21.1)
Apfel score, *n* (%)	
0	17 (11.2)
1	45 (29.6)
2	76 (50)
3	14 (9.2)
Comorbidities, *n* (%)	
Diabetes mellitus	27 (17.8)
Hypertension	33 (21.7)
Coronary artery disease	9 (5.9)
Pulmonary disease	15 (9.9)
Chronic kidney disease	2 (1.3)
Thyroid disease	15 (9.9)
Cirrhosis	4 (2.6)
Medication use, *n* (%)	
Oral antidiabetic agents	21 (13.8)
Biguanides (metformin)	18 (11.8)
Dipeptidyl peptidase-4 (DPP-4) inhibitors	6 (3.9)
Sodium–glucose cotransporter-2 (SGLT-2) inhibitors	6 (3.9)
Sulfonylureas	2 (1.3)
Thiazolidinediones (TZDs)	4 (2.6)
Insulin	5 (3.3)
Antihypertensive agents	32 (21.1)

ASA: American Society of Anesthesiologists, BMI: Body mass index, PONV: Postoperative nausea and vomiting, SD: Standard deviation. VAS: Visual analog scale.

**Table 2:** Macroscopic diagnosis and medications used for sedation.

Macroscopic Diagnosis, *n* (%)	
Antral gastritis	2 (1.3)
Pangastritis	79 (52)
Gastroesophageal reflux	32 (21.1)
Hiatal hernia	6 (3.9)
Peptic ulcer	14 (9.2)
Celiac disease	4 (2.6)
Malignancy	1 (0.7)
Candida esophagitis	1 (0.7)
Esophageal varices	6 (3.9)
Alkaline reflux gastritis	7 (4.6)
**Drugs,** ***n*** **(%)**	
Propofol	151 (99.3)
Midazolam	34 (22.4)
Fentanyl	13 (8.6)
Remifentanil	2 (1.3)
Ketamine	1 (0.7)

**Table 3:** Postoperative nausea and/or vomiting.

Preoperative nausea or vomiting, *n* (%)	27 (17.8)
Postoperative nausea, *n* (%)	13 (8.6)
Postoperative vomiting, *n* (%)	0 (0)
Need for antiemetic treatment, *n* (%)	2 (1.3)

**Table 4:** Variables between participants who experienced PONV and those who did not.

	PONV (+) (*n* = 13)	PONV (−) (*n* = 139)	*p*-Value
Age (years) (mean ± SD)	51.6 ± 12.2	44.5 ± 14.4	0.088 ^a^
Gender, *n* (%) ^b^			0.020 ^c^
Female	13 (100)	97 (69.8)	
Male	0 (0)	42 (30.2)	
Weight (kg) median (min–max)	77 (54–105)	73 (40–115)	0.239 ^d^
Height (cm) median (min–max)	160 (145–165)	163 (145–194)	0.027 ^d^
BMI median (min–max)	31.6 (22.5–42.0)	26.6 (16–51)	0.009 ^d^
BMI category			
<30	4 (30.8)	103 (74.1)	0.002 ^c^
≥30	9 (69.2)	36 (25.9)	
ASA score median (min–max)	2 (1–3)	2 (1–3)	0.287 ^d^
VAS scores median (min–max)	0 (0–8)	0 (0–10)	0.087 ^d^
Smoking status, *n* (%) ^b^	2 (15.4)	40 (28.8)	0.517 ^c^
Alcohol consumption, *n* (%) ^b^	-	7 (5.0)	1.000 ^c^
History of PONV, *n* (%) ^b^	4 (30.8)	15 (10.8)	0.060 ^c^
Motion sickness, *n* (%) ^b^	4 (30.8)	28 (20.1)	0.474 ^c^
Apfel score, *n* (%) ^b^			0.008 ^c^
0	-	17 (12.2)	
1	-	45 (32.4)	
2	11 (84.6)	65 (46.8)	
3	2 (15.4)	12 (8.6)	
Complaint of nausea or vomiting, *n* (%) ^b^	7 (53.8)	20 (14.4)	0.002 ^c^
Comorbidities, *n* (%) ^b^			
Diabetes mellitus	5 (38.5)	22 (15.8)	0.056 ^c^
Hypertension	5 (38.5)	28 (20.1)	0.157 ^c^
Coronary artery disease	0 (0)	9 (6.5)	1.000 ^c^
Pulmonary disease	2 (15.4)	13 (9.4)	0.619 ^c^
Chronic kidney disease	0 (0)	2 (1.4)	1.000 ^c^
Thyroid disease	5 (38.5)	10 (7.2)	0.004 ^c^
Cirrhosis	0 (0)	4 (2.9)	1.000 ^c^
Medication use, *n* (%) ^b^			
Oral antidiabetic agents	6 (46.2)	15 (10.8)	0.003 ^c^
Metformin use	5 (38.5)	13 (9.4)	0.009 ^c^
Insulin	0 (0)	5 (3.6)	1.000 ^c^
Antihypertensive agents	4 (30.8)	28 (20.1)	0.474 ^c^
Macroscopic diagnosis, *n* (%) ^b^			0.561 ^c^
Antral gastritis	-	2 (1.4)	
Pangastritis	7 (53.8)	72 (51.8)	
Gastroesophageal reflux	3 (23.1)	29 (20.9)	
Hiatal hernia	1 (7.7)	5 (3.6)	
Peptic ulcer	-	14 (10.1)	
Celiac disease	-	4 (2.9)	
Malignancy	-	1 (0.7)	
Candida esophagitis	-	1 (0.7)	
Esophageal varices	-	6 (4.3)	
Alkaline reflux gastritis	2 (15.4)	5 (3.6)	
Drugs, *n* (%) ^b^			
Propofol	13 (100)	138 (99.3)	1.000 ^c^
Midazolam	1 (7.7)	33 (23.7)	0.299 ^c^
Fentanyl	2 (15.4)	11 (7.9)	0.307 ^c^
Remifentanyl	-	2 (1.4)	1.000 ^c^
Ketamine	-	1 (0.7)	1.000 ^c^
Duration of procedure (min), median	7 (7–8)	7 (6–8)	0.73 ^d^

^a^: Student’s *t* test, ^b^: Column percentage, ^c^: Fischer’s exact test, ^d^: Mann–Whitney U test. ASA: American Society of Anesthesiologists, BMI: Body mass index, PONV: Postoperative nausea and vomiting, SD: Standard deviation, VAS: Visual analog scale.

## Data Availability

The data presented in this study are available on request from the corresponding author. The data are not publicly available due to patient privacy concerns and ethical restrictions.

## Abbreviations

ASA	American Society of Anesthesiologists
BMI	Body Mass Index
EGD	Esophagogastroduodenoscopy
PACU	Post-Anesthesia Care Unit
PDNV	Post-discharge Nausea and Vomiting
PONV	Postoperative Nausea and Vomiting
VAS	Visual Analog Scale
